# Effect of Post-Processing Heat Treatment on Micro-Contact Damage of Zirconia-Reinforced Lithium Silicate Dental Materials

**DOI:** 10.3390/ma17091961

**Published:** 2024-04-24

**Authors:** José A. Pérez, Fernando Rodríguez-Rojas, Óscar Borrero-López, Estíbaliz Sánchez-González

**Affiliations:** Departamento de Ingeniería Mecánica, Energética y de los Materiales, Universidad de Extremadura, 06006 Badajoz, Spain; joseperez@unex.es (J.A.P.); keko@unex.es (F.R.-R.); oborlop@unex.es (Ó.B.-L.)

**Keywords:** dental materials, lithium silicate, microstructure, contact damage, fracture

## Abstract

Zirconia-reinforced lithium silicate (ZLS) is utilized as a material for prosthetic tooth crowns, offering enhanced strength compared to other dental glass-ceramics. In this study, we investigate a commercial ZLS material, provided in a fully crystallized form. We examine the effects of an optional post-processing heat treatment on micro-contact damage using controlled indentation tests simulating the primary modes of contact during chewing: axial and sliding. Our findings indicate that the heat treatment does not affect mechanical properties such as the elastic modulus, hardness and indentation fracture toughness. However, it does enhance the resistance to contact damage by fracture and chipping in both axial and sliding modes, as well as the resistance to crack initiation measured from sliding tests. This improvement is attributed to the refinement of the flaw population achieved through the heat treatment. The results are analysed using principles of contact and fracture mechanics theory, discussing their significance in prosthetic dentistry.

## 1. Introduction

Modern dental prosthetic crowns predominantly utilize ceramic materials and their composites, chosen based on aesthetic and mechanical requirements, often involving tradeoffs. Among available options, dental zirconias are renowned for their superior strength and wear resistance, albeit potentially presenting challenges in machinability and aesthetics compared to less robust materials such as glass-ceramics or ceramic-polymer composites [1,2,3,4,5,6,7,8,9,10,11].

**Figure 1:**
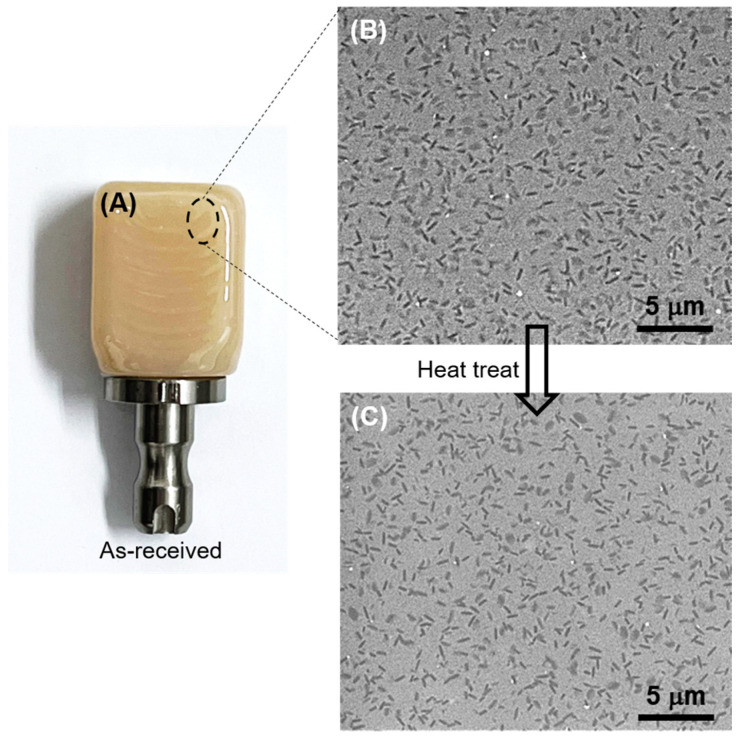
(**A**) Photograph of an as-received ZLS CAD/CAM block. SEM images of the microstructure of ZLS dental glass-ceramics (from [14]): (**B**) as-received, and (**C**) heat-treated.

Zirconia-reinforced lithium silicate (ZLS) materials represent an advancement in dental glass-ceramics, offering enhanced strength and durability while maintaining excellent aesthetics [12]. This is achieved by incorporating 10% zirconium oxide in the glass matrix to bolster strength, and by limiting the size of lithium silicate crystallites [13]. Commercial ZLS dental materials are typically sourced in blocks (Figure 1A) suitable for computer-aided design/computer-aided manufacturing (CAD/CAM) dentistry. According to the manufacturer’s specifications [13], one of such ZLS materials can be employed in as-received, already fully-crystallized condition which possesses a natural tooth colour. An optional heat treatment (post-processing) is recommended to further enhance flexural and biaxial fracture strength by approximately ~150/160 MPa without changing the aesthetics.

A gap in the literature exists regarding the systematic examination of how post-processing treatments may impact the contact damage (e.g., fracture-induced chipping) of ZLS dental materials, beyond conventional mechanical property measurements. This is relevant because dental prosthetic crowns experience cyclic contacts against opposing dentition during chewing, involving a complex combination of axial and lateral forces, occasional bite overloads, and exposure to aggressive media where micron-sized abrasive particles may be present [6,15,16]. Mechanical degradation in the form of fracture and wear in turn has the potential to compromise the performance, strength and durability of the crown. Notably, the ability to resist mechanical degradation does not necessarily correlate with single-valued mechanical properties as measured from standardized tests [8].

**Figure 2:**
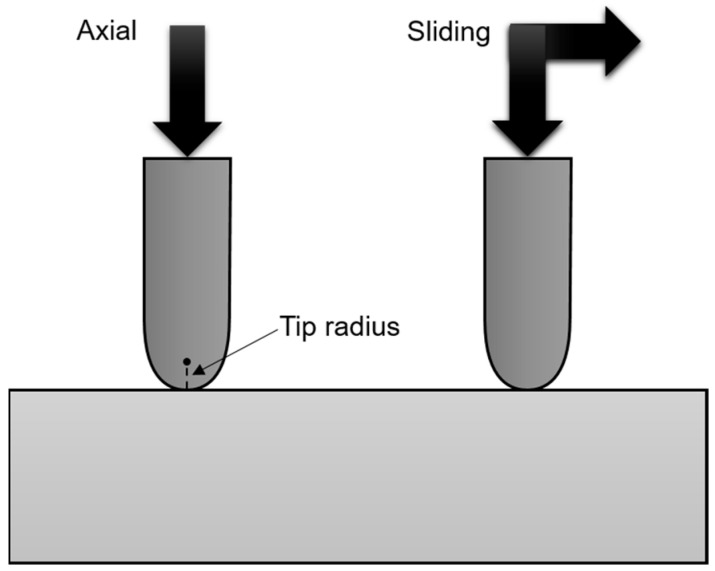
Schematic (cross-sectional detail) of the axial and sliding contact configurations simulated by indentation tests. Not at scale.

To address this gap, we conduct a materials engineering investigation of the fundamental contact damage of as-received (untreated) and heat-treated commercial ZLS glass-ceramics using controlled in vitro indentation tests, in both axial and sliding configurations (Figure 2). The effects of contact/abrasive size are evaluated using tips of varying radii. Results are analysed with respect to the materials’ microstructure (structure-property), within the framework of contact fracture mechanics. Furthermore, we briefly discuss the implications for prosthetic dentistry, with a view to providing insights for the selection of dental materials.

## 2. Experimental Procedure

### 2.1. Materials Processing

The ZLS materials used were sourced from market-available CAD/CAM blocks (Celtra Duo HT, Dentsply Sirona, NC, USA). Plane parallel specimens for testing of thickness ≈ 1.2–1.5 mm were prepared by mechanical sawing from as-supplied (untreated) blocks. A dental furnace (Multimap Touch, Dentsply, PA, USA) was employed to heat-treat half of the specimens in a dental prosthetics laboratory (Clínica David Maestre, Valverde de Leganés, and LAB Dental, Badajoz, Spain), according to the manufacturer’s specifications: first heating to 500 °C (hold 3 min), continued by heating to 820 °C at 60 °C/min (hold 1 min), and finally gradual cool-down with the chamber closed until 500 °C, when furnace doors are opened [7].

The top surfaces of all specimens (as-received and heat-treated) were gently ground with fine sandpaper, lapped (30 μm), and polished with diamond suspensions using the same routine, consisting of the following steps: 15 μm (10 min), 9 μm (10 min), 6 μm (10 min), 3 μm (15 min), and 1 μm (20 min). As a result, the surface roughness of all specimens is expected to be below 1 μm.

### 2.2. Microstructural Characterization

The structure and fracture surface of the materials (non-metallized) were observed at the microstructural scale by scanning electron microscopy (FE-SEM; Quanta 3D FEG, FEI, Rotterdam, The Netherlands) employing secondary and backscattered electrons at intermediate accelerating voltages (10–20 kV).

### 2.3. Mechanical Characterization

To measure contact mechanical properties, the following standard indentation tests were performed at room temperature in air: (i) Hertzian tests (5535, Instron, Canton, MA, USA), on gold-coated samples, applying loads of 15–1500 N with WC spherical indenters of radii 7.94 and 4.76 mm. The sizes of the residual imprints were measured by optical microscopy (Epiphot 300, Nikon, Tokyo, Japan) and, following established procedures [17], stress-strain curves were constructed. For each material, the Young’s modulus (*E*) was calculated from the linear part of the stress-strain curve [17,18]. (ii) Vickers tests (MV-1, Matsuzawa, Japan), with a diamond tip applying a 9.8 N load. For each material, 10 indentations were performed to estimate the hardness (*H*) and fracture toughness (*K_IC_*) from the dimensions (measured by optical microscopy) of the residual imprints, applying standard theory [18,19].

Advanced indentation experiments were conducted to study the micro-contact damage. Tests were performed at room temperature in air, using computer-controlled equipment (Revetest RST3, Anton Paar, Graz, Austria) with Rockwell-C (sphero-conical), with diamond indenters of radii 200 μm and 20 μm. Tests were performed in both axial and sliding loading configurations (see Figure 2). In the axial tests, a series of pre-tests were performed at 50 N/min, increasing the maximum load at prescribed intervals of 20 N. The test surfaces were subsequently examined by optical microscopy to identify the load range within which chipping first occurs. Such intervals were then probed in more detail, using loading steps between 1 N and 5 N. Each test was replicated ×3. Surfaces were again inspected after testing to accurately determine critical chipping loads. The criterion used was that the critical chipping load was the smallest load for which all 3 of the 3 indentations at such load showed a well-formed chip particle. Tests were also performed in sliding mode, ×3 for each material and tip dimension, using a sliding speed of 5 mm/min, at gradually increasing normal load from an initial value of 1 N (loading rate 100 N/min with the large tip; 25 N/min with the small tip). Test surfaces were subsequently inspected by optical microscopy. For each test, critical chipping load was calculated from the corresponding microscopy image using the sliding speed and loading rate (more details in [6]).

Sliding tests using the larger tip (200 µm) were also employed to estimate the resistance to sliding fracture (σ*) of the materials. Tests were conducted in progressive loading using: initial normal load 1 N, final normal load of 25 N, loading rate 25 N/min, and sliding rate 3 mm/min. *N* = 25 tests were performed for each material. After each scratch test, the residual imprint was examined in detail using the optical microscope to determine the critical fracture (normal) load (*F_C_*) and the contact radius (*a*) at the onset of cracking, as well as the coefficient of friction (*f*) logged by the equipment at that point. For each material, the σ* values were obtained from the experimental (*F_C_*, *a*, *f*) values using a contact mechanics model [20]. These were then ranked in ascending order, and a probability of failure value (*P_f_*) was allocated to each one of them, using *P_f_* = (*i* − 0.5)/*N*, for the *i*-th value. A two-parameter Weibull probability function was used to fit the data, from which the characteristic resistance to sliding fracture (*σ*_0_*) and Weibull modulus (*m*) were determined [21] (more details in [22]).

### 2.4. Stress Simulations

In selected cases, the software package FilmDoctor^®^ (studio istress version 1.0.0.1, SIO^®^, Saxonian Institute of Surface Mechanics, Ruegen, Germany) was employed to simulate contact stresses. From the contact conditions and the materials’ elastic property values, the software calculates the elastic stress field in single-layer and multilayer systems, applying the extended Hertzian model [23].

## 3. Results and Discussion

### 3.1. Microstructure

Figure 1B,C show SEM micrographs representative of the microstructure of as-received and heat-treated ZLS dental glass-ceramics, respectively. At the microstructural scale, no significant differences can be detected between the two. Both microstructures exhibit a distribution of lithium-based crystals within a zirconia silicate glassy matrix. Crystal phase content is ≈50 vol%, and their compositions are known to be primarily Li_2_SiO_3_, followed by Li_2_Si_2_O_5_ and Li_3_PO_4_ [13,24,25]. Crystal sizes range from nanometric, beyond the current resolution, to micrometric. The majority of observed crystals appear rod-shaped, with an average size of 0.7 ± 0.1 μm and an aspect ratio of 3.5 ± 0.6 [7]. Hence, based on the SEM observations, the optional post-processing heat treatment does not significantly alter the relative phase content and crystallinity of the ZLS investigated materials. It is worth noting that this contrasts with other commercially available ZLS materials, which are supplied in pre-crystallized form and require a crystallization treatment to achieve both aesthetic and strength requirements [25].

### 3.2. Mechanical Properties

The contact-mechanical properties of the ZLS materials investigated also appear to remain unaffected by the post-processing heat treatment [26]. Elastic modulus, hardness and indentation fracture toughness values of 99 ± 6 GPa, 6.2 ± 0.2 GPa, and 0.70 ± 0.03 MPa·m^1/2^, respectively, were measured through Hertzian and Vickers tests in both as-received and heat-treated materials. In comparison to other dental glass-ceramics, ZLS materials generally exhibit improved mechanical properties, except for fracture toughness [4,6,7,22]. Lithium disilicate glass-ceramics, with their larger, interconnected crystals, demonstrate significantly higher toughness [7,22]. It is important to note that, while the Vickers indentation method may be considered less accurate than other techniques, it proves suitable for measuring the fracture toughness of the ZLS materials investigated and assessing any potential differences. This is because, under the load applied in our tests, the materials exhibit the required response, characterized by clean radial cracks observed on the surface [27,28].

### 3.3. Contact Damage

**Figure 3:**
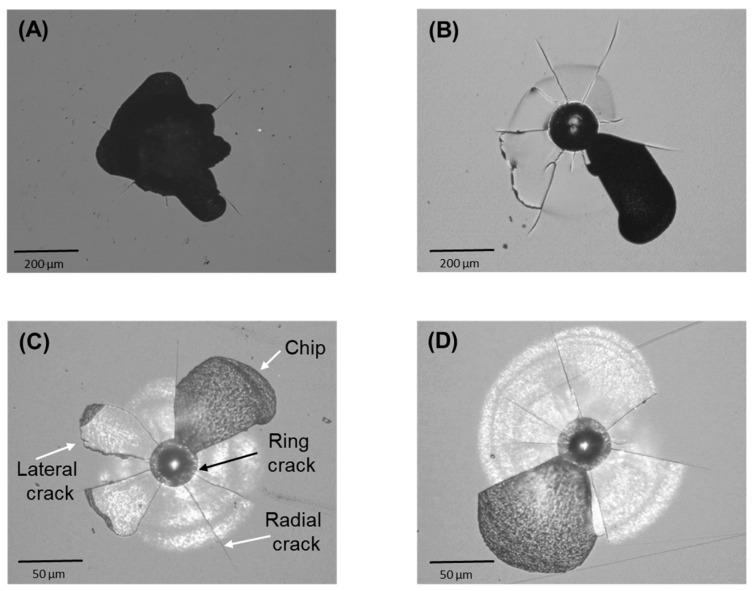
Optical micrographs representative of the damage generated by axial contacts of different size in ZLS glass-ceramics: (**A**) as-received, tip radius 200 μm, load 115 N; (**B**) heat-treated, tip radius 200 μm, load 130 N; (**C**) as-received, tip radius 20 μm, load 10 N; (**D**) heat-treated, tip radius 20 μm, load 12 N.

Controlled indentation tests with sphero-conical tips in both axial and sliding modes enable simulations of the fundamental modes of dental contact. Results from such tests are typically more reproducible than those from chewing simulators, and facilitate assessing subtle microstructural effects. Figure 3 presents micrographs illustrating the damage generated by axial contacts of different size (radius 200 μm and 20 μm) on both as-received and heat-treated ZLS glass-ceramics. When the applied load is sufficiently high, the concentrated stresses induce different fracture modes around the indentation site, including ring-like cracks around the circular contact, radial cracks perpendicular to the loading axis, and sub-surface lateral cracks (some reaching the surface). Of particular relevance in dentistry is the phenomenon of *chipping*, characterized by the wholesale dislodgement of material fragments (chips) due to the coalescence of cracks (typically radial and lateral) [29]. The loads required for the onset of fracture and chipping decrease with decreasing tip radius from 200 μm to 20 μm. It is noteworthy that, in contrast with the previous single-value mechanical properties, the critical chipping load is influenced by the heat treatment. With the larger tip radius (200 μm), the critical chipping load for the as-received material was measured at 115 ± 5 N, while for the heat-treated material it was 130 ± 5 N—a 13% increase. Similarly, with the smaller tip radius (20 μm), the critical chipping loads measured in as-received and heat-treated materials were 10 ± 1 N and 12 ± 1 N, respectively, representing a 20% increase.

**Figure 4:**
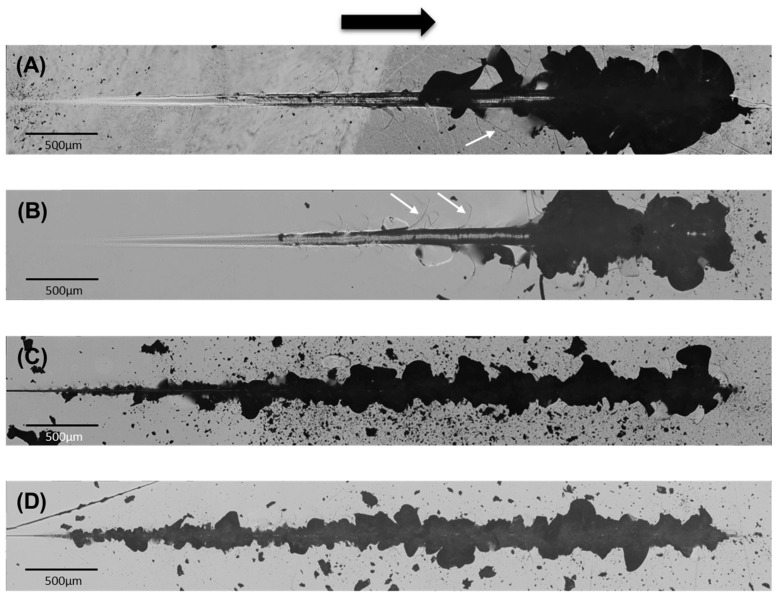
Optical micrographs (panoramic) representative of the damage generated by sliding contacts of different size (progressive loading, from 1 N to 100 N with tip radius 200 μm and from 1 N to 25 N with tip radius 20 μm) in ZLS glass-ceramics: (**A**) as-received, tip radius 200 μm with white arrows pointing at radial cracks (from [6]); (**B**) heat-treated, tip radius 200 μm with white arrows pointing at radial cracks; (**C**) as-received, tip radius 20 μm (from [6]); (**D**) heat-treated, tip radius 20 μm. The black arrow indicates the direction of sliding.

Figure 4 presents panoramic micrographs of the damage resulting from sliding contacts of different size, at progressively increasing normal load, on the investigated ZLS glass-ceramics. With the larger tip (200 μm), low initial loads lead to surface smearing and deformation, evolving into fracture at intermediate loads. The first fracture mode is partial ring cracks, perpendicular to the sliding direction. Subsequent sliding leaves behind a series of parallel cracks in the wake of the contact, with progressively decreasing spacing. At higher loads, radial cracks extend outwards from the scratch track at an angle to the sliding direction. Ultimately, the coalescence of radial and partial ring/cone cracks results in material removal through chipping. As in the axial case, the loads required for the onset of fracture and chipping decrease with decreasing tip radius from 200 μm to 20 μm, with the heat treatment influencing the critical loads. With the larger tip (200 μm), the measured critical fracture and chipping loads were 5.4 ± 0.1 N and 54 ± 4 N (as-received), and 7.2 ± 0.1 N and 64 ± 4 N (heat-treated). With the smaller tip (20 μm), the critical fracture and chipping loads were 2.2 ± 0.1 N and 2.5 ± 0.3 N (as-received), and 2.5 ± 0.1 N and 2.7 ± 0.3 (heat-treated).

**Figure 5:**
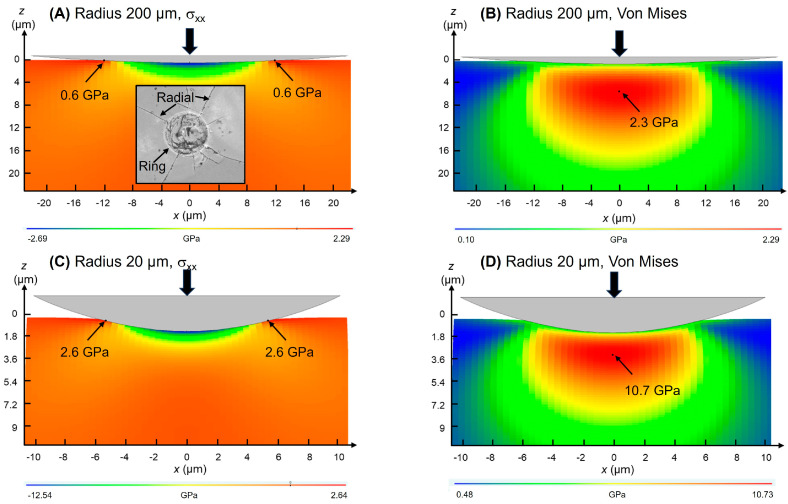
FilmDoctor^®^ simulations of the relevant components of the contact stress field induced on a model isotropic, elastic layer of elastic modulus 99 GPa (simulating ZLS) by a sliding contact (diamond indenter) at a reference applied load (axial) of 1 N: (**A**) tensile stress *σ*_xx_, indenter radius 200 μm; (**B**) Von Mises stress, indenter radius 200 μm; (**C**) tensile stress *σ*_xx_, indenter radius 20 μm; (**D**) Von Mises stress, indenter radius 20 μm. Cartesian coordinates are used, with z being the normal loading direction. The contour plots show the stress at the xz cross-section. Positive/negative values indicate tensile/compressive stress. Note the different *x*- and *z*-axis scales used in each graph. The inset in (**A**) is an optical microscopy detail of the cracking modes generated by the stress field on the contact surface.

The fracture modes observed can be explained based on the analysis of the main components of the stress field generated by axial and sliding contacts. Figure 5 shows simulations of the cross-sectional distribution of normal and Von Mises (proxy for maximum shear [30]) stresses, induced by axial contacts of radii 200 μm and 20 μm, at a low reference load (1 N). The maximum tensile stress is located on the surface, right outside the contact circle. Because the maximum tensile stresses have radial direction, when their magnitude exceeds a critical value, they open ring-shaped cracks, as shown in the inset of Figure 5A (high magnification detail) [17]. On the other hand, the maximum shear stress (Figure 5B) is located below the surface, approximately at ~0.5 *a*, with *a* being the Hertzian contact radius, and is responsible for plastic and quasi-plastic deformation processes. Upon unloading, the residual elastic-plastic indentation stress field propagates some of the sub-surface microcracks previously generated, which eventually reach the contact surface with radial orientation (i.e., radial cracks, Figure 5A inset). Depending on the specific micro-crack orientation, propagation may be parallel to the contact surface, developing lateral cracks as observed, for example, in Figure 3C [17,18,19]. The simulations indicate that the greater pressures attained using smaller tips translate into an increase in both the maximum surface tensile stress and the sub-surface Von Mises stress with decreasing contact size (Figure 5C,D). This explains, qualitatively, the lower loads required for chipping using the 20 μm tip compared to the 200 μm tip.

**Figure 6:**
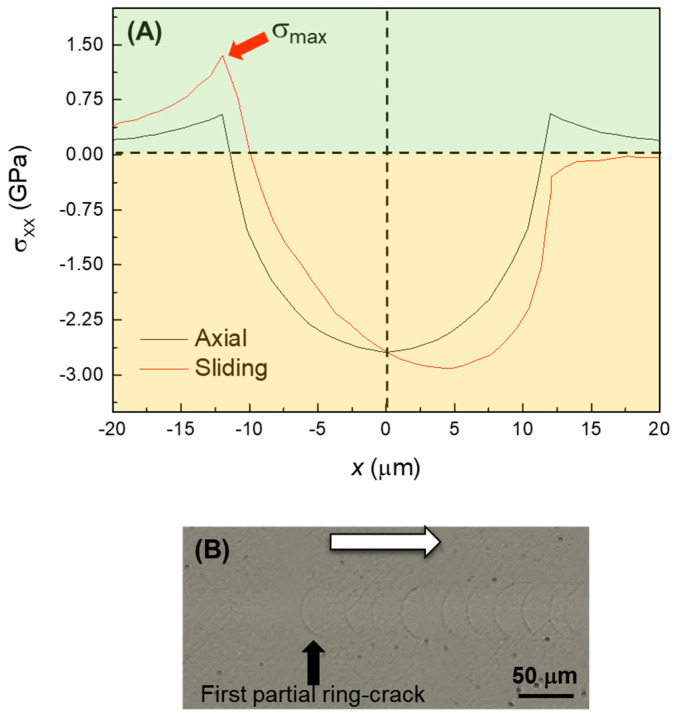
(**A**) 2-D plot at the contact surface (*z* = 0 μm) of the tensile stress *σ*_xx_ (for a reference load 1 N) on a model ZLS layer simulated by FilmDoctor^®^ for purely axial (black line) and sliding (*f* = 0.2, red line) contact. Cartesian coordinates are used, with *z* being the normal loading direction, and *x* the direction of sliding. The green and yellow regions correspond to tensile and compressive stresses, respectively. Dashed line separate these regions. (**B**) Optical micrograph showing the onset of partial ring-cracking in a ZLS material upon sliding with a 200 μm tip at progressively increasing load.

The addition of a lateral force upon sliding has a relatively minor effect on the Von Mises stress—it basically shifts the maximum slightly ahead of the normal axis [20]. However, it has a significant impact on the stress at the contact surface. In particular, friction increases the maximum tensile stress at the trailing half of the contact, while the stress at the leading half becomes compressive, as observed in the line graphs (Figure 6) of the normal stress distributions on the surface for the 200 μm tip (axial/frictionless vs. sliding/friction) [20]. This asymmetry of the maximum contact stress leads to the initial opening not of fully developed ring cracks, but of half rings (i.e., only in the rear edge of the contact, the so-called partial ring/cone cracks) [31]. A detail of such partial ring cracks upon sliding contact can be observed in Figure 6B. As in the axial case, subsequent increases in the applied load lead to the formation of radial cracks. In the sliding case, the moving tensile component of the stress field pulls the radial cracks forward, which explains the angle with respect to the sliding direction (e.g., white arrows in Figure 4A,B). Decreasing the contact size also translates into an increase of the magnitude of the stresses, which again explains the lower chipping loads measured with the 20 μm tip compared to the 200 μm tip. In fact, as a result of the greater contact pressure even at low loads, partial ring cracks are not observed with the smaller tip, and the initial fracture mode is radial cracking followed by chipping at low loads.

### 3.4. Resistance to Sliding Fracture

**Figure 7:**
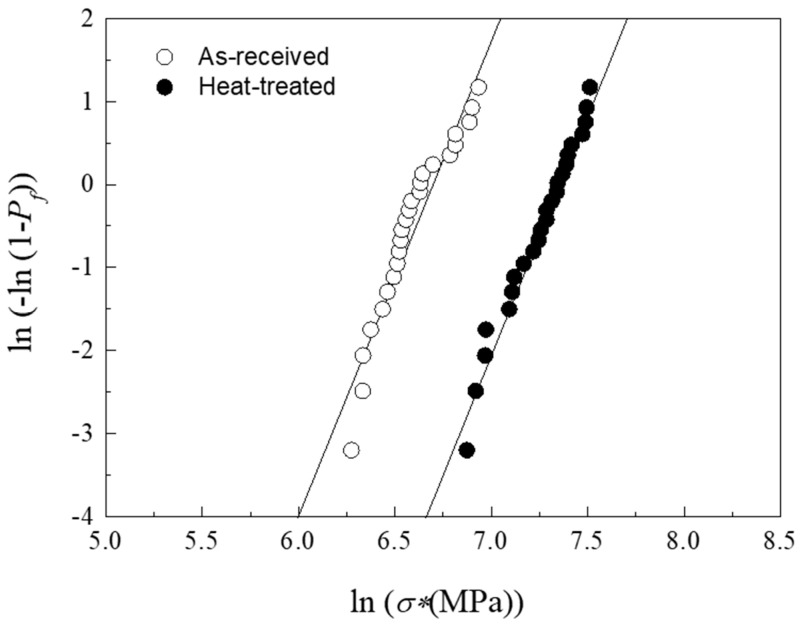
Weibull plot of probability of failure (*P_f_*) as a function of fracture stress (σ*) after sliding tests with a 200 μm tip on as-received and heat-treated ZLS glass-ceramics. Circles represent experimental data and solid lines are best linear fits to each data set.

The fact that the initial fracture mode of the ZLS materials upon scratching with the larger 200 μm tip is that of partial ring cracks allows this test to be employed to assess the resistance to sliding fracture. The theoretical framework is provided by the expression from Hamilton and Goodman for the maximum tensile stress at the rear edge of a sliding Hertzian contact [20]:

(1) $$\sigma_{max}=P_{0}\left(\frac{1-2\upsilon }{3}+\frac{4+\upsilon }{8}f\right)$$

where *P*_0_ and *f* are the applied Hertzian pressure and coefficient of friction at that contact point, and *υ* the Poisson’s ratio of the material. Thus, measurement of the critical load *F_C_* and *f* at which the first partial ring crack appears (data collected by the equipment) allows an estimation of the material’s resistance to sliding fracture (σ*) using (1). To deal with the intrinsic variability of the fracture phenomenon in brittle materials, several tests were performed in as-received and heat-treated ZLS materials and analysed using Weibull statistics [22]. The results are shown in Figure 7, where *P_f_* represents the probability of failure. As with the critical chipping loads, the post-processing heat treatment has a positive impact on the resistance to sliding fracture. However, in this case the extent of the improvement is significantly greater, with the heat-treated material showing a characteristic resistance *σ*_0_* = 1587.6 MPa and Weibull modulus/reliability *m* = 5.8 vs. *σ*_0_* = 804.3 MPa and *m* = 5.7 in the as-received material. 

### 3.5. General Discussion

**Figure 8:**
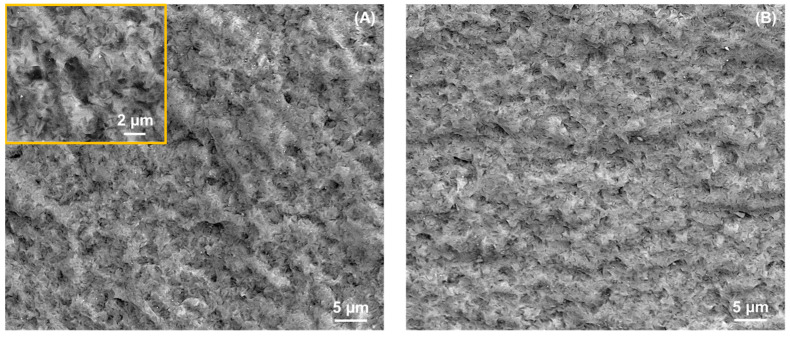
SEM micrographs representative of the fracture surface of ZLS glass-ceramics: (**A**) as-received, with inset corresponding to a higher magnification detail; (**B**) heat-treated.

Within the limitations of the current study (chiefly, the relatively low sample size in the indentation tests, and the low material diversity as only one commercial material was investigated), the results obtained demonstrate that the optional post-processing heat treatment applied to the ZLS material investigated does not significantly alter its single-value mechanical properties (*E*, *H*, *K_IC_*). However, it does increase the critical loads for chipping under any given contact configuration (axial, sliding) and size, as well as the sliding fracture strength. In essence, this treatment makes the material more resistant to the initiation of cracks, and to material removal by crack coalescence processes. Given that the heat treatment does not significantly affect the observed crystallinity of the glass-ceramics (Figure 1), this effect is attributed to a refinement of the flaw population. Such defects are traditionally challenging to detect on polished surfaces such as the ones in Figure 1 [32,33], but they become apparent on fracture surfaces, as illustrated in Figure 8. The SEM images reveal the presence of defects, such as µm-sized voids [19], in both the as-received and the heat-treated materials. However, the frequency and size of these defects appear to be relatively smaller after the heat treatment. According to Griffith’s fracture mechanics relation (*σ*_F_~*c*^−1/2^, with *c* representing the size of the critical defect precursor for fracture [18,19]), a reduction in the average defect size can in turn lead to an increase in the fracture strength of the heat-treated material without compromising aesthetics, aligning with the manufacturer’s specifications [13].

### 3.6. Implications and Future Studies

This study has significant implications in prosthetic dentistry and may aid in selecting and developing materials with improved strength and durability. Although the post-processing heat treatment for ZLS glass-ceramics involves greater processing cost and time, it results in improved mechanical stability of the prosthesis without sacrificing aesthetics. Similar treatments could be considered for other types of dental materials. Furthermore, the findings emphasize that single-value mechanical properties such as elastic modulus, hardness, and fracture toughness may not adequately reflect prosthesis durability, adding to previous evidence obtained from wear tests on biphasic dental materials [8]. Material removal processes, such as chipping, involving complex interactions between different fracture modes, are best assessed by considering both mechanical properties and microstructural features (e.g., weak interfaces, defects).

Subsequent investigations should delve into the effects of heat treatments on additional mechanical properties of interest, including flexural strength (both in the bulk and on the margin interface [34]) and contact fatigue. Furthermore, follow-up studies should employ chewing simulators for greater realism, and broaden the scope to encompass other commercial dental ZLS and glass-ceramics systems. These efforts should aim to develop innovative heat treatments that yield the optimal combination of properties.

## 4. Conclusions

We have investigated the effect of a post-processing heat treatment on the damage generated by micrometric axial and sliding contacts in ZLS dental glass-ceramics. Based on the results and analyses, the following conclusions can be drawn:

1. The heat treatment does not significantly alter the crystallinity of the ZLS glass-ceramics, but it does reduce the frequency and size of processing flaws compared to the untreated material.
2. Within experimental errors, the heat treatment does not impact the elastic modulus, hardness, and indentation fracture toughness of the materials.
3. The heat treatment leads to an increase in critical loads for fracture and chipping in both axial and sliding contacts, with critical loads decreasing as the contact size decreases.
4. A significant improvement in fracture strength, as measured from scratch tests, is observed with statistical (Weibull) significance following the heat treatment.
5. The enhanced resistance to contact damage can be attributed to the refinement of the flaw population achieved through the heat treatment.

## Data Availability

Data are contained within the article.
